# Executive functioning but not IQ or illness severity predicts occupational status in bipolar disorder

**DOI:** 10.1186/s40345-019-0168-6

**Published:** 2020-02-28

**Authors:** Julia Drakopoulos, Timea Sparding, Caitlin Clements, Erik Pålsson, Mikael Landén

**Affiliations:** 1 Department of Psychiatry and Neurochemistry, Institute of Neuroscience and Physiology, The Sahlgrenska Academy, University of Gothenburg, Sahlgrenska University Hospital, Blå Stråket 15, 413 45 Gothenburg, Sweden; 2 Department of Psychology, University of Pennsylvania, 3720 Walnut Street, Philadelphia, PA 19104 USA; 3 Department of Medical Epidemiology and Biostatistics, Karolinska Institutet, Stockholm, Sweden; 4 Center for Autism Research, The Children’s Hospital of Philadelphia, 2716 South St, Philadelphia, PA 19104 USA

**Keywords:** Employment, Observational study, Neuropsychological tests, Bipolar disorder, Cognitive dysfunction, Executive function

## Abstract

**Background:**

Bipolar disorder is associated with significant functional deficits including occupational functioning. Despite the high rates of unemployment and sick leave in the patient population, only a limited number of studies have examined factors associated with occupational functioning in bipolar disorder. The aim of the study was to investigate the relative importance of demographic, clinical, and neuropsychological factors on occupational dysfunction in bipolar disorder.

**Methods:**

A sample of 120 partially or fully remitted bipolar disorder I and II patients were included in the study. Patients were stratified into an active and an inactive group based on the number of hours per week working or studying. Active (n = 86) and inactive (n = 34) patients were compared with respect to demographic factors, clinical characteristics, medication, measures of psychosocial functioning, and cognitive functioning (i.e., IQ and executive functions). No other cognitive domains were examined.

**Results:**

Univariate analyses revealed better overall cognitive function in active patients in terms of IQ and executive functioning. However, only executive functioning accounted for a significant amount of the variance in occupational status when other significant predictors were taken into account.

**Conclusions:**

Executive functioning was a more powerful predictor of occupational status in bipolar disorder patients than IQ and other clinical factors, including illness severity.

## Background

Bipolar disorder is one of the leading causes of disability worldwide, which incurs higher costs on the health care system and society than most other illnesses (Murray and Lopez 1997). The bulk of societal costs of bipolar disorders are due to indirect costs such as sick-leave, unemployment, and early retirement (Ekman et al. 2013). This is because as many as 30 to 60% of patients with bipolar disorder do not regain full occupational or social functioning after illness onset (MacQueen et al. 2001). Functional recovery also lags behind recovery from clinical symptoms and may be incomplete despite the absence of mood symptoms (Goldberg et al. 1995).

Employment is an important area of functioning. Factors previously associated with poor occupational or functional outcome in bipolar disorder include the number of affective episodes (MacQueen et al. 2000; Zimmerman et al. 2010), lingering subsyndromal depression (Dickerson et al. 2004; Bonnín et al. 2010; Burdick et al. 2010), history of psychiatric hospitalizations (Dickerson et al. 2004; Burdick et al. 2010; Gutiérrez-Rojas et al. 2011), total symptom severity (Goetza et al. 2007), age (Depp et al. 2012), educational attainment (Reed et al. 2010), age at onset (Perlis et al. 2009), history of psychotic symptoms (Tohen et al. 1990, 2000), and comorbid psychiatric conditions such as panic disorder and substance abuse (Hajek et al. 2005; Zimmerman et al. 2010).

Cognitive performance is another domain that has been linked to poor occupational functioning (Mur et al. 2009; Gilbert and Marwaha 2013; Ryan et al. 2013; Bonnín et al. 2014). Executive functions seem to be particularly important for psychosocial functioning and practical tasks of daily life (Lezak 2012). Bipolar disorder patients feature cognitive impairments (mainly deficits in attention, verbal memory, and executive functioning) on group level that persist during euthymia (Robinson et al. 2006; Mur et al. 2007; Palsson et al. 2013; Sparding et al. 2015a, b; Salarvan et al. 2019).

It is important to establish factors influencing occupational functioning in order to tailor and optimize care management in bipolar disorder. Previous research has linked clinical, demographic, and cognitive factors to occupational functioning, but their relative influence remains unclear. The aim of the current study was to investigate the relative importance of demographic, clinical and neuropsychological factors on occupational dysfunction in bipolar disorder.

## Methods

### Subjects

The present study is part of the St. Göran Bipolar Project, which is an interdisciplinary, prospective, longitudinal study of bipolar disorder conducted in Sweden. Patients were recruited and examined at the Northern Stockholm Mental Health Service and followed over time. The methods of this study have been outlined in detail previously (Rydén et al. 2009a; b; Ekman et al. 2010). Briefly, patients were examined at a time when the patient was considered stable with respect to mood symptoms by the treating physician. This means that patients were not in an acute depressive or hypomanic episode, but not always completely asymptomatic. Patients were diagnosed using the Affective Disorder Evaluation (ADE) and the Mini International Neuropsychiatric Interview (M.I.N.I.) (Sheehan et al. 1998). The ADE is a semi-structured interview that was employed in the Systematic Treatment Enhancement Program of Bipolar Disorder (STEP-BD) project (Sachs et al. 2003). It includes an adapted version of the affective module of the Structural Clinical Interview for DSM-IV (SCID). The M.I.N.I. was used to screen for other psychiatric disorders. A final best estimate diagnosis was determined by a consensus panel of experienced psychiatrists. Bipolar disorder severity was rated by an experienced clinician using the Clinical Global Impression (CGI) rating scale (Guy 1976). To screen for alcohol and substance abuse, patients completed two self-report questionnaires: the Alcohol Use Disorders Identification Test (AUDIT) (Saunders et al. 1993) and the Drug Use Disorders Identification Test (DUDIT) (Berman et al. 2005). Data on medication were collected from either the baseline diagnostic assessment or the somatic examination, depending on which appointment occurred closest in time to the neuropsychological testing.

The retirement age for the guaranteed pension scheme in Sweden is 65 years. The current study therefore included participants aged 18 to 64 years who met DSM-IV criteria for bipolar I or II disorder (n = 120). Only data for individuals with ≤ 14 on the Montgomery-Åsberg Depression Rating Scale (MADRS) (Asberg and Schalling 1979) and the Young Ziegler Mania Rating Scale (YMRS) (Young et al. 1978) were included in the present study. The reason for choosing 14 as the cut off on MADRS and YMRS was to strike a balance between excluding patients with manifest mood symptoms while keeping patients with subsyndromal lingering symptoms, which is prevalent in bipolar disorder (Judd et al. 2002).

There were no cases of intellectual disability, nor any somatic disorders of clinical significance that might affect cognitive performance (such as stroke or other neurological disorders) in this cohort. Patients with current alcohol or substance dependency were excluded, but patients with a history of alcohol or substance abuse were retained in order to study the natural course of the disorder and to obtain a representative clinical sample.

The project was approved by Stockholm Regional Ethical Review Board, and all participants provided written and oral informed consent.

### Neuropsychological assessments

In the overarching St. Göran bipolar study, the participants completed a broad neuropsychological test battery (Sparding et al. 2015a, b) to assess cognitive functioning. For the purpose of this study, we preselected the *Wechsler Adult Intelligence Scale III* as a measure of general cognitive ability (Wechsler 1997), and the *Delis*–*Kaplan Executive Function System* (Delis et al. 2001) as measures of executive functions. No other cognitive domains were examined.

The Wechsler Adult Intelligence Scale (WAIS-III) is the most widely used intelligence quotient (IQ) test and is composed of 10 verbal and non-verbal performance tests: Information, Similarities, Vocabulary, Arithmetic, Digit Span, Block Design, Matrix Reasoning, Picture Completion, Digit Symbol-Coding, and Symbol Search. The subtests generate a measure of general intelligence (mean value = 100; standard deviation = 15). The Vocabulary subtest is frequently used as proxy for premorbid IQ (Martinez-Aran et al. 2007; Ryan et al. 2013; Bonnín et al. 2014).

The Delis–Kaplan Executive Function System (D-KEFS) is a normed and standardized set of nine subtests used to evaluate key components of executive function, including mental flexibility, concept formation, problem solving, and inhibition. Most subtests include one baseline task (condition 1) followed by more difficult tasks/conditions. The latter tasks are considered to be the primary measures of executive functioning. We used five individual tests from the D-KEFS:

i. The *Color*-*Word Interference Test,* which corresponds to the Stroop Color Word Test and measures the ability to inhibit automatic verbal responses, in this case by reading names of colors (e.g., red, blue, green) printed in incongruent font colors. The test conditions are: (1): Color Naming, (2): Word Reading, (3): Inhibition, and (4): Inhibition/Switching.
ii. The *Verbal Fluency Test* measures the ability to produce as many words as possible from a given category in 60 s. The categories are either phonemic (i.e., words beginning with a specified letter) or semantic (i.e., objects such as animals or fruit). Test results provide information about language skills and verbal processing ability, as well as problem solving and inhibition. The test conditions are: (1): Letter Fluency, (2): Category Fluency, and (3): Category Switching.
iii. The *Design Fluency Test* evaluates the subjects’ ability to draw as many different designs as possible in 60 s by connecting a set of printed dots. The executive functions required for the test include the capacity of initiating problem-solving behavior, cognitive flexibility, and inhibition of automatic responses. The test conditions are: (1): Filled Dots, (2): Empty Dots Only, (3): Switching.
iv. The *Trail Making Test* (TMT) contains measures of cognitive flexibility, visual attention, and motor speed. The test requires the subject to connect a sequence of 25 consecutive numbers and/or letters as fast as possible. In TMT 2, the subject connects a number sequence only. In TMT 4, which is similar to TMT B, the subject alternates between numbers and letters (e.g., 1-A, 2-B, etc.). The test conditions are: (1): Visual scanning, (2): Number Sequencing, (3): Letter Sequencing, (4): Number-Letter Switching, and (5): Motor Speed.
v. The Tower Test assesses planning and spatial problem-solving abilities such as the ability to inhibit perseverative and impulsive responses. Visual attention and visual-spatial ability are fundamental skills required for this task.

### Assessment of occupational status

We had access to categorized information on occupational functioning. The alternatives were (i) working or studying more than 50% time in a competitive employment (or educational) setting; (ii) working or studying in a competitive employment (or educational) setting 50% time or less (which in Sweden usually means receiving early retirement benefit, or temporary or permanent sick-leave benefits at ≥ 50%), (iii) unemployed, (iv) supported employment, (v) idle. The sample was divided into two groups according to their current occupational status: active versus inactive. Active patients (n = 86) included the highest functioning group including subjects who held a competitively-obtained occupation, or were university students, at > 50% (i.e., working/studying more than 20 h per week). Inactive patients (n = 34) included all the other options, i.e., subjects who were unemployed, received early retirement benefit, or were on temporary or permanent sick-leave at ≥ 50% (i.e., working/studying 20 h or less per week). This group also included subjects who held supported employments (i.e., outside the open labor market), or were enrolled in vocational rehabilitation programs.

### Assessment of psychosocial functioning

We used scores from both the symptom and function domains of the Global Assessment of Functioning (GAF) Scale (Luborsky 1975) to assess overall functioning. The Sheehan Disability Scale (Sheehan 1983) was administered to obtain information about patients’ subjective assessment of psychosocial dysfunction following their illness. This scale is a composite of three self-rated items designed to assess the extent to which three major areas in life are compromised by psychiatric symptomatology. The patient rates the extent to which his or her (1) work or school, (2) social life or leisure activities, and (3) home life or family responsibilities are impaired by his or her symptoms on a 10-point visual analogue scale (0 = not at all, 1–3 = mildly, 4–6 = moderately, 7–9 = markedly, 10 = extremely).

### Statistical procedures

Data analyses were carried out with the statistical package IBM SPSS 23.0 for Windows (IBM Corp. Armonk, NY). We first examined which variables were different between the groups of active and inactive patients. Between-group differences in demographic and clinical characteristics were evaluated with univariate analyses of variance (ANOVAs) by examining a single factor of the group (active vs. inactive). Pearson’s Chi-square tests were run for dichotomous and categorical variables.

For cognitive measures, Analyses of Covariance (ANCOVAs) were performed for each test to investigate differences between the active and inactive group. Effect sizes were calculated with the formula for partial eta squared (η^2^). Current medication with benzodiazepines was used as a covariate because a significantly higher proportion of patients in the inactive group were prescribed such drugs. Scaled scores were used as outcome measures because they account for group differences in age. Two-tailed tests were performed for all analyses, and statistical significance was defined as p < 0.05.

A composite measure of executive function was created using principal component analysis (PCA) using the SIMCA software (SIMCA-P 13.0 software, Umetrics AB, Umeå, Sweden). A PCA reduces data dimensions and summarizes systematic variation in a model by aggregating variables in ‘components’ or ‘latent variables’ that describe the correlations structure (Eriksson et al. 2013). By creating a PCA-model, it is possible to (i) examine measures that are highly correlated, (ii) look at all the executive tests together without losing any test specific information, (iii) avoid problems with mass significance. A total of 11 subtest scores were selected from five D-KEFS tests: the *Tower Test*, the *Verbal Fluency Test*, the *Color Word Test*, the *Design Fluency Test*, and the *Trail Making Test.* All test conditions were included, except the baseline tasks and non-executive measures (i.e., TMT Motor Speed, which did not differ between the groups [*p *= 0.112]). 18 subjects (12 active, 6 inactive) were automatically excluded from the analysis as they had completed less than 50% of the tests. Two parameters are important to consider when evaluating a principal component: goodness of fit (R2), which expresses the explained variation, and goodness of prediction (Q2), which expresses the power to anticipate data, either internally or externally (Eriksson et al. 2013).

The first principal component (PC1) was thereafter used as an executive functioning score as a predictor in the binary logistic regression models to predict occupational status. Each test’s loading (P1) reveals how much each test contributes to the latent variable, executive functioning. We conducted three successive logistic regressions to estimate the relative influence of executive functions, IQ, and clinical variables to predict occupational status (active vs. inactive). In the first model, only executive functioning (PC1) was used as predictor variable. In the second model, IQ was added. The active and the inactive group differed in age, but we did not add age and sex into the logistic regression model because the cognitive variables (IQ and the PC of executive functioning) were in scaled scores already adjusted for age and sex. In the third model, we added clinical variables that differed significantly between groups in the univariate analyses: history of psychotic symptoms, history of involuntary psychiatric care (i.e., being sectioned under the Mental Health Act), and current benzodiazepine use. All clinical variables were categorical and coded 1 or 0 (“yes” = 1, “no” = 0). Variance of inflation tests performed to control for multicollinearity did not reveal conflicting relations between the covariates.

## Results

The characteristics of the 86 active and 34 inactive patients are displayed in Table 1. Subjects in the inactive group were slightly older than the active group. A significantly larger proportion of the inactive patients had a history of psychotic symptoms, and had at least one involuntary hospitalization (i.e., had been sectioned under the Mental Health Act). The use of benzodiazepines was more prevalent in the inactive group than the active group (44% vs. 20%, p < 0.05). No other group differences were observed with respect to comorbid conditions, bipolar subtype, age at illness onset, subsyndromal symptomatology (as measured with YMRS and MADRS), number of previous depressive or manic episodes, or bipolar disorder severity as rated with CGI. The inactive patients showed worse functioning as measured by the GAF symptom and function scales, as well as all three dimensions of the Sheehan Disability Scale.

**Table 1:** Demographic, clinical, functional, and pharmacological variables

	Active (n = 86)	Inactive (n = 34)	Statistics F	*p*-value
Demographics, mean (SD)				
Age, years	35.3 (10.8)	40.0 (13.5)	4.01	0.048
Clinical features, mean (SD)				
Age at onset, years	16.2 (3.8)	16.4 (4.6)	0.07	0.793
Age at first therapy, years	26.7 (8.0)	24.6 (10.7)	1.14	0.288
Total number of manic episodes	2.0 (3.6)	2.4 (3.1)	0.35	0.556
Total number of depressive episodes	9.2 (14.0)	13.3 (15.1)	2.03	0.157
MADRS score	3.4 (3.2)	4.0 (3.8)	0.54	0.463
YMRS score	1.7 (2.4)	2.1 (3.0)	0.50	0.483
GAF symptom score	69.7 (9.7)	63.1 (9.0)	11.54	0.001
GAF function score	69.7 (9.7)	63.9 (9.5)	8.63	0.004
CGI (total)	4.5 (0.9)	4.6 (1.2)	0.41	0.524
Sheehan disability scale^a^, mean (SD)				
Work/school	4.2 (3.4)	7.1 (3.4)	14.41	< 0.001
Social life	3.6 (3.2)	5.6 (2.7)	7.71	0.007
Family life/home responsibilities	2.6 (2.9)	5.2 (2.3)	17.80	< 0.001

	Active (n = 86)	Inactive (n = 34)	χ^2^	*p*-value
Sex, no. (%)			0.57	0.451
Female	52 (60.5)	18 (52.9)		
Male	34 (39.5)	16 (47.1)		
Diagnosis, no. (%)			1.09	0.297
Bipolar I disorder	54 (63.5)	25 (73.5)		
Bipolar II disorder	31 (36.5)	9 (26.5)		
University studies	52 (61.2)	15 (44.1)	2.87	0.090
Comorbidity, no. (%)				
ADHD	11 (15.7)	6 (22.2)	0.57	0.450
Anxiety disorder^b^	15 (17.4)	10 (29.4)	2.12	0.146
Clinical features, no. (%)				
Lifetime history of psychotic symptoms	43 (50.6)	24 (70.6)	3.95	0.047
History of involuntary psychiatric care	38 (44.7)	22 (64.7)	3.87	0.049
History of alcohol abuse	16 (19.5)	9 (26.5)	0.69	0.407
History of substance abuse	9 (10.8)	6 (18.2)	1.13	1.129
Type of current medication, no. (%)				
Lithium	53 (61.6)	20 (58.8)	0.08	0.777
Antidepressants	32 (37.2)	15 (44.1)	0.49	0.485
Anticonvulsants	24 (27.9)	15 (44.1)	2.92	0.088
Antipsychotics	23 (26.7)	9 (26.5)	0.00	0.976
Central stimulants	2 (2.3)	3 (8.8)	2.58	0.108
Benzodiazepines	17 (19.8)	15 (44.1)	7.39	0.007

*MADRS* Montgomery-Åsberg Depression Rating Scale, *YMRS* Young Ziegler Mania Rating Scale, *GAF* Global Assessment of Functioning Scale, *CGI* Clinical Global Impression Rating Scale

^a^Sheehan Disability Scale is a self-report scale in which patients rate to what extent their functioning in three different life areas is impaired by psychiatric symptomatology (0 = not at all, 1–3 = mildly, 4–6 = moderately, 7–9 = markedly, 10 = extremely)

^b^Anxiety disorders include the following DSM-IV diagnoses: generalized anxiety disorder, panic disorder, agoraphobia, social anxiety disorder, obsessive–compulsive disorder, and post-traumatic stress disorder

The ANCOVAs showed differences between the active and inactive group in the majority of cognitive tests, where active patients performed better overall. Active patients also showed a significantly higher IQ than inactive patients (109 vs. 101, *p *= 0.014).

The PCA model created with 11 D-KEFS executive functioning subtests was significant (R2X = 0.611; Q2X = 0.415). The executive functioning subtests loaded robustly on the first principal component (R2 = 0.464; Q2 = 0.329; Table 2).

**Table 2:** Neurocognitive assessment for all participants

		Active	Inactive	Statistics F	d.f.	*p*-value	η^2^
WAIS-III							
Full scale IQ		109.0 (1.6)	101.3 (2.6)	6.311	1	0.014	0.06
Premorbid IQ (vocabulary)		11.8 (0.3)	10.9 (0.4)	3.086	1	0.082	–
D-KEFS							
Principal component (PC)							
Test included in PC1	Loading on PC1 (P1)						
Color word 3	0.325713	10.3 (0.3)	7.6 (0.6)	16.225	1	< 0.001	0.14
Color word 4	0.326118	10.0 (0.3)	8.4 (0.6)	6.057	1	0.016	0.06
Verbal fluency 2	0.340796	13.0 (0.5)	10.9 (0.8)	5.010	1	0.027	0.05
Verbal fluency 3—correct responses	0.334285	11.8 (0.4)	10.1 (0.6)	4.944	1	0.028	0.05
Verbal fluency 3—switching accuracy	0.30624	12.1 (0.4)	9.6 (0.6)	12.161	1	0.001	0.10
Design fluency 2	0.22314	11.4 (0.4)	10.2 (0.6)	2.542	1	0.114	–
Design fluency 3	0.26376	11.3 (0.3)	9.9 (0.5)	5.028	1	0.027	0.05
TMT 2	0.303005	9.7 (0.4)	7.6 (0.6)	7.415	1	0.008	0.07
TMT 3	0.309674	10.0 (0.3)	8.0 (0.6)	8.206	1	0.005	0.08
TMT 4	0.330988	9.7 (0.3)	7.9 (0.6)	7.438	1	0.008	0.07
Tower test	0.221951	11.2 (0.4)	9.4 (0.7)	4.360	1	0.040	0.05

Results from WAIS-III and D-KEFS subtests are scaled scores, for which the average range is 9-11 (low average scores = 7–8; high average scores = 12–13). The average range for WAIS-III Full Scale IQ is 85–115

*WAIS-III* Wechsler Adult Intelligence Scale, *D-KEFS* Delis–Kaplan Executive Function System

Three logistic regressions were run to evaluate the relative influence of executive functioning in predicting occupational status (active vs. inactive). In the first regression model (Table 3), we only used our composite measure of executive functioning as predictor. It demonstrated that the odds of a person to be in the inactive group decreased by 36% as the principal component score increased by 1.

**Table 3:** Logistic regression predicting likelihood of an inactive occupational status (executive functioning as only predictor)

	*p*-value	OR	95% CI
Executive functions	< 0.001	0.64	0.51–0.80
Constant	< 0.001	0.31	

Occupational status as a dichotomized dependent variable (inactive = 1; active = 0). Executive functions (composite measure) as the only covariate. Cox and Snell’s R^2^ = 0.17. Nagelkerke’s R^2^ = 0.25. The model correctly classified 73.5% of the cases, with 91.9% of the active patients and 25.0% of the inactive patients correctly classified, n = 102

The second logistic regression (Table 4) was used to evaluate the additional impact of IQ. IQ did not make a unique statistically significant contribution to the model (*p *= 0.183) despite a considerably higher rate of correctly classified inactive patients in this model compared to the previous one (42.3% vs. 25.0%).

**Table 4:** Logistic regression predicting likelihood of an inactive occupational status (executive functioning and IQ as predictors)

	*p*-value	OR	95% CI
Executive functions	< 0.001	0.53	0.38–0.76
WAIS-III–IQ	0.183	1.04	0.98–1.10
Constant	0.090	0.01	

Occupational status as a dichotomized dependent variable (inactive = 1; active = 0). Executive functions (composite measure) and Full Scale IQ (measured with WAIS-III) as covariates. A test of the full model with the two predictors against the constant-only model was significant (omnibus Chi-square = 21.05, df = 2, *p* < 0.001.), Cox and Snells R^2^ = 0.20. Nagelkerke’s R^2^ = 0.29. The model correctly classified 81.1% of patients (95.7% of the active patients and 42.3% of the inactive patients were correctly classified, n = 95

The third logistic regression was used to evaluate the additional impact of both IQ and clinical factors. No clinical variables made a statistically significant contribution to the model (*p* values > 0.05). Only executive function significantly predicted occupational status (*p *= 0.001). The odds of a person to be in the inactive group decreased by 45% as each principal component score increased by 1. None of the other variables made a unique, significant contribution to the model (Table 5).

**Table 5:** Logistic regression predicting likelihood of an inactive occupational status (cognitive and clinical factors as predictors)

	*p*-value	OR	95% CI
Executive functions	0.001	0.55	0.38–0.78
WAIS-III–IQ	0.196	1.04	0.98–1.10
History of psychosis	0.240	0.41	0.09–1.81
Involuntary care	0.245	2.46	0.54–11.2
Benzodiazepines	0.224	0.47	0.14–1.58
Constant	0.135	0.01	

Occupational status as a dichotomized dependent variable (inactive = 1; active = 0) and five covariates, including Executive functions (composite measure) and Full Scale IQ (measured with WAIS-III). Categorical covariates are prior history of psychotic symptoms (yes = 1; no = 0), history of involuntary care (i.e. if ever sectioned under the Mental Health Act; yes = 1; no = 0), and benzodiazepines (yes = 1; no = 0). The full model significantly predicted an inactive occupational status (omnibus Chi-square = 24.11, df = 5, *p* < 0.001). Cox and Snell’s R^2^ = 0.23 Nagelkerke’s R^2^ = 0.33. The model correctly classified 78.7% of the patients (92.6% of the active patients and 42.3% of the inactive patients were correctly classified), n = 94

## Discussion

The high rate of occupational dysfunction is a major issue in bipolar disorder. Here we studied the relative influence of clinical, demographic, and cognitive factors on occupational status by comparing bipolar disorder patients that worked or studied (active group) with those who did not (inactive group). We found that executive functioning was a stronger determinant of occupational functioning than general cognitive functioning (IQ) and other important clinical factors, including illness severity. In fact, executive functioning was the only factor that accounted for a significant amount of the variance in occupational status when clinical factors and IQ was taken into account. Taken together, our results suggest that poor executive functioning is the main factor associated with occupational dysfunction in bipolar disorder patients.

When individual predictors were considered separately, our findings align with previous cross-sectional and longitudinal studies that assessed the relationship with occupational status in bipolar disorder. First, psychotic symptoms during affective episodes and hospitalizations have been linked to a worse occupational and functional outcome in previous studies (Dickerson et al. 2004; Burdick et al. 2010; Gutiérrez-Rojas et al. 2011). We find similar differences regarding both lifetime history of psychotic symptoms and previous hospitalizations between the active and the inactive group. Second, general cognitive ability has been strongly associated with occupational status in both clinical and control samples (Schmidt and Hunter 2004). In line with this, we found in univariate analyses that the active group had significantly higher IQ than inactive patients. The groups did not, however, differ regarding premorbid IQ. Third, previous studies have found an association between executive functioning test scores and occupational status in bipolar disorder (Mur et al. 2008; Baune and Malhi 2015) similar to our findings. Finally, we found a higher prescription of benzodiazepines in the inactive patients.

Importantly, however, we demonstrate that none of the above variables significantly predict occupational status above and beyond the influence of executive functions. In terms of cognitive functioning, it was particularly notable that IQ was not a significant predictor of occupational status when compared to the model with executive function alone, further demonstrating the strong relationship between executive functioning and occupational status in bipolar disorder.

Instead, cognitive flexibility and inhibitory control as measured by condition 3 of the Color Word Test, the Verbal Fluency Test, and the Trail Making Test captured the largest differences between active and inactive patients (η^2^ = 0.08–0.14). Cognitive flexibility is needed to adjust thinking and behavior in response to changing demands, and inhibitory control function facilitates inhibition of a dominant response in favor of one that is consistent with a long-term goal (Delis et al. 2001). Tests measuring these executive processes also stood out in previous studies predicting the occupational status of bipolar disorder patients (Ryan et al. 2013; Bonnín et al. 2014; O’Donnell et al. 2017).

The construct of executive functioning generally describes the integrated cognitive processes responsible for planning, initiating, sequencing, and monitoring complex goal-directed behavior (Royall et al. 2002). Different measures of executive function generally correlate strongly with each other but less with IQ (Friedman et al. 2006). Executive impairments are considered to be more disabling than other cognitive deficits as they affect many aspects of behavior (Lezak 2012). Despite this, the ecological validity of executive functioning performance tests has been questioned by ADHD researchers, who have found that self-report ratings were better predictors of occupational impairment (Barkley and Murphy 2010). The fact that our study clearly captured executive deficits in the inactive group suggests that executive function test scores do reflect real-life executive impairments and can be useful in clinical assessment.

The finding that occupationally inactive bipolar disorder patients manifest executive cognitive deficits has clinical implications. Early identification these impairments could give valuable information about the individuals’ ability to return to his/her work, and guide which possible adaptions could be made in the workplace. Some cognitive remediation programs have been developed and shown promising results on occupational and social outcomes (Sanchez-Moreno et al. 2017).

### Strengths and limitations

Strengths of this study include that we defined occupational functioning by means of actual time working (or studying), which provides a hard outcome of functioning. Our study also involved a meticulous clinical characterization, including well-established neuropsychological tests. The participants were representative of bipolar disorder patients receiving psychiatric care, as nearly all patients with this diagnosis in the catchment area were referred to the Bipolar Affective Disorder unit for treatment by the time of enrollment. Finally, our study complements the literature with data from a Scandinavian cohort in a field where most studies have been conducted by research groups in the US and Spain.

We also acknowledge a number of limitations. First, it should be noted that 72% of the subjects were occupationally active, which is a rather high number compared with previous reports (MacQueen et al. 2001). A possible explanation is that the catchment area of the recruiting clinic includes districts in Stockholm where the unemployment rates are lower than in the rest of the country, and the mean incomes above the Swedish average. Second, our analyses did not investigate the relative prediction of executive functioning compared to other cognitive functions previously found to predict occupational status in bipolar disorder patients, such as verbal memory (Dickerson et al. 2004), visual memory (Mur et al. 2009), and processing speed (Burdick et al. 2010). Future studies should further explore the relative contributions of these factors. Third, a potential source of bias is that 12 active and 6 inactive participants were excluded from the PCA because they had completed less than 50% of the tests. However, the proportion of excluded participants did not differ between the groups (Fisher exact test statistic value = 0.3585, p > 0.05). Fourth, the difference between measures of premorbid IQ and Full Scale IQ may indicate that the inactive group has declined in cognitive functioning. Our study did not investigate whether such decline predicted occupational status. The last and most important limitation is the cross-sectional design. We cannot determine if executive dysfunction causes occupational disability or if unemployment worsens executive functioning.

## Conclusions

Bipolar disorder patients with worse executive functioning are more likely to be occupationally inactive. These deficits can be identified with neuropsychological tests and should be targeted in treatment and rehabilitation.

## Data Availability

The data that support the findings of this study are available on reasonable request from the corresponding author (TS). The data are not publicly available due to information that could compromise research participant privacy.

## References

[CR1] Asberg M, Schalling D (1979). Construction of a new psychiatric rating instrument, the Comprehensive Psychopathological Rating Scale (CPRS). Prog Neuropsychopharmacol..

[CR2] Barkley RA, Murphy KR (2010). Impairment in occupational functioning and adult ADHD: the predictive utility of executive function (EF) ratings versus EF tests. Arch Clin Neuropsychol.

[CR3] Baune BT, Malhi GS (2015). A review on the impact of cognitive dysfunction on social, occupational, and general functional outcomes in bipolar disorder. Bipolar Disord.

[CR4] Berman AH, Bergman H, Palmstierna T, Schlyter F (2005). Evaluation of the drug use disorders identification test (DUDIT) in criminal justice and detoxification settings and in a Swedish population sample. Eur Addict Res.

[CR5] Bonnín CM, Martínez-Arán A, Torrent C, Pacchiarotti I, Rosa AR, Franco C (2010). Clinical and neurocognitive predictors of functional outcome in bipolar euthymic patients: a long-term, follow-up study. J Affect Disord.

[CR6] Bonnín CM, Torrent C, Goikolea JM, Reinares M, Solé B, Valentí M (2014). The impact of repeated manic episodes and executive dysfunction on work adjustment in bipolar disorder. Eur Arch Psychiatry Clin Neurosci.

[CR7] Burdick KE, Goldberg JF, Harrow M (2010). Neurocognitive dysfunction and psychosocial outcome in patients with bipolar I disorder at 15-year follow-up. Acta Psychiatr Scand.

[CR8] Delis DC, Kaplan E, Kramer JH (2001). Delis–Kaplan executive function system (D-KEFS) examiner’s manual.

[CR9] Depp CA, Mausbach BT, Bowie C, Wolyniec P, Thornquist MH, Luke JR (2012). Determinants of occupational and residential functioning in bipolar disorder. J Affect Disord.

[CR10] Dickerson FB, Boronow JJ, Stallings CR, Origoni AE, Cole S, Yolken RH (2004). Association between cognitive functioning and employment status of persons with bipolar disorder. Psychiatr Serv.

[CR11] Ekman CJ, Lind J, Rydén E, Ingvar M, Landén M (2010). Manic episodes are associated with grey matter volume reduction a voxel-based morphometry brain analysis. Acta Psychiatr Scand.

[CR12] Ekman M, Granstrom O, Omerov S, Jacob J, Landen M (2013). The societal cost of bipolar disorder in Sweden. Soc Psychiatry Psychiatr Epidemiol.

[CR13] Eriksson L, Byrne T, Johansson E, Trygg J, Wikström C (2013). Multi- and megavariate data analysis: basic principles and applications.

[CR14] Friedman NP, Miyake A, Corley RP, Young SE, Defries JC, Hewitt JK (2006). Not all executive functions are related to intelligence. Psychol Sci.

[CR15] Gilbert E, Marwaha S (2013). Predictors of employment in bipolar disorder: a systematic review. J Affect Disord.

[CR16] Goetza I, Tohen M, Reed C, Lorenzo M, Vieta E (2007). Functional impairment in patients with mania: baseline results of the EMBLEM study. Bipolar Disord.

[CR17] Goldberg JF, Harrow M, Grossman LS (1995). Course and outcome in bipolar affective disorder: a longitudinal follow-up study. Am J Psychiatry.

[CR18] Gutiérrez-Rojas L, Jurado D, Gurpegui M (2011). Factors associated with work, social life and family life disability in bipolar disorder patients. Psychiatry Res.

[CR19] Guy W. ECDEU Assessment Manual for Psychopharmacology. Rockville, MD, US Department of Health, Education, and Welfare Public Health Service Alcohol, Drug Abuse, and Mental Health Administration. 1976.

[CR20] Hajek T, Slaney C, Garnham J, Ruzickova M, Passmore M, Alda M (2005). Clinical correlates of current level of functioning in primary care-treated bipolar patients. Bipolar Disord.

[CR21] Judd LL, Akiskal HS, Schettler PJ, Endicott J, Maser J, Solomon DA (2002). The long-term natural history of the weekly symptomatic status of bipolar I disorder. Arch Gen Psychiatry.

[CR22] Lezak MD (2012). Neuropsychological assessment.

[CR23] Luborsky L (1975). Clinicians’ judgments of mental health: specimen case descriptions and forms for the health-sickness rating scale. Bull Menninger Clin.

[CR24] MacQueen GM, Young LT, Robb JC, Marriott M, Cooke RG, Joffe RT (2000). Effect of number of episodes on wellbeing and functioning of patients with bipolar disorder. Acta Psychiatr Scand.

[CR25] MacQueen GM, Young LT, Joffe RT (2001). A review of psychosocial outcome in patients with bipolar disorder. Acta Psychiatr Scand.

[CR26] Martinez-Aran A, Vieta E, Torrent C, Sanchez-Moreno J, Goikolea JM, Salamero M (2007). Functional outcome in bipolar disorder: the role of clinical and cognitive factors. Bipolar Disord.

[CR27] Mur M, Portella MJ, Mártinez-Arán A, Pifarré J, Vieta E (2007). Persistent neuropsychological deficit in euthymic bipolar patients: executive function as a core deficit. J Clin Psychiatry..

[CR28] Mur M, Portella MJ, Martínez-Arán A, Pifarré J, Vieta E (2008). Long-term stability of cognitive impairment in bipolar disorder: a 2-year follow-up study of lithium-treated euthymic bipolar patients. J Clin Psychiatry.

[CR29] Mur M, Portella MJ, Martinez-Aran A, Pifarre J, Vieta E (2009). Influence of clinical and neuropsychological variables on the psychosocial and occupational outcome of remitted bipolar patients. Psychopathology.

[CR30] Murray CJ, Lopez AD (1997). Alternative projections of mortality and disability by cause 1990–2020: global Burden of Disease Study. Lancet.

[CR31] O’Donnell LA, Deldin PJ, Grogan-Kaylor A, McInnis MG, Weintraub J, Ryan KA (2017). Depression and executive functioning deficits predict poor occupational functioning in a large longitudinal sample with bipolar disorder. J Affect Disord.

[CR32] Palsson E, Figueras C, Johansson AG, Ekman CJ, Hultman B, Ostlind J (2013). Neurocognitive function in bipolar disorder: a comparison between bipolar I and II disorder and matched controls. BMC Psychiatry..

[CR33] Perlis RH, Dennehy EB, Miklowitz DJ, Delbello MP, Ostacher M, Calabrese JR (2009). Retrospective age at onset of bipolar disorder and outcome during two-year follow-up: results from the STEP-BD study. Bipolar Disord.

[CR34] Reed C, Goetz I, Vieta E, Bassi M, Haro JM (2010). Work impairment in bipolar disorder patients—results from a two-year observational study (EMBLEM). Eur Psychiatry..

[CR35] Robinson LJ, Thompson JM, Gallagher P, Goswami U, Young AH, Ferrier IN (2006). A meta-analysis of cognitive deficits in euthymic patients with bipolar disorder. J Affect Disord.

[CR36] Royall DR, Lauterbach EC, Cummings JL, Reeve A, Rummans TA, Kaufer DI (2002). Executive control function: a review of its promise and challenges for clinical research: a report from the committee on research of the American Neuropsychiatric Association. J Neuropsychiatry Clin Neurosci.

[CR37] Ryan KA, Vederman AC, Kamali M, Marshall D, Weldon AL, McInnis MG (2013). Emotion perception and executive functioning predict work status in euthymic bipolar disorder. Psychiatry Res.

[CR38] Rydén E, Johansson C, Blennow K, Landén M (2009). Lower CSF HVA and 5-HIAA in bipolar disorder type 1 with a history of childhood ADHD. J Neural Transm.

[CR39] Rydén E, Thase ME, Stråht D, Åberg-Wistedt A, Bejerot S, Landén M (2009). A history of childhood attention-deficit hyperactivity disorder (ADHD) impacts clinical outcome in adult bipolar patients regardless of current ADHD. Acta Psychiatr Scand.

[CR40] Sachs GS, Thase ME, Otto MW, Bauer M, Miklowitz D, Wisniewski SR (2003). Rationale, design, and methods of the Systematic Treatment Enhancement Program for bipolar disorder. Biol Psychiatry.

[CR41] Salarvan S, Sparding T, Clements C, Ryden E, Landen M (2019). Neuropsychological profiles of adult bipolar disorder patients with and without comorbid attention-deficit hyperactivity disorder. Int J Bipolar Disord..

[CR42] Sanchez-Moreno J, Martinez-Aran A, Vieta E (2017). Treatment of functional impairment in patients with bipolar disorder. Curr Psychiatry Rep..

[CR43] Saunders JB, Aasland OG, Babor TF, de la Fuente JR, Grant M (1993). Development of the alcohol use disorders identification test (AUDIT): WHO Collaborative Project on early detection of persons with harmful alcohol consumption-II. Addiction.

[CR44] Schmidt FL, Hunter J (2004). General mental ability in the world of work: occupational attainment and job performance. J Pers Soc Psychol.

[CR500] Sheehan DV (1983). The anxiety disease.

[CR45] Sheehan DV, Lecrubier Y, Sheehan KH, Amorim P, Janavs J, Weiller E (1998). The Mini-International Neuropsychiatric Interview (M.I.N.I.): the development and validation of a structured diagnostic psychiatric interview for DSM-IV and ICD-10. J Clin Psychiatry..

[CR46] Sparding T, Silander K, Pålsson E, Östlind J, Sellgren C, Ekman CJ (2015). Cognitive functioning in clinically stable patients with bipolar disorder I and II. PLoS ONE.

[CR47] Sparding T, Silander K, Pålsson E, Östlind J, Sellgren C, Ekman CJ (2015). Cognitive functioning in clinically stable patients with bipolar disorder I and II. PLoS ONE.

[CR48] Tohen M, Waternaux CM, Tsuang MT (1990). Outcome in Mania. A 4-year prospective follow-up of 75 patients utilizing survival analysis. Arch Gen Psychiatry.

[CR49] Tohen M, Hennen J, Zarate CM, Baldessarini RJ, Strakowski SM, Stoll AL (2000). Two-year syndromal and functional recovery in 219 cases of first-episode major affective disorder with psychotic features. Am J Psychiatry.

[CR50] Wechsler D (1997). WAIS-III administration and scoring manual.

[CR51] Young RC, Biggs JT, Ziegler VE, Meyer DA (1978). A rating scale for mania: reliability, validity and sensitivity. Br J Psychiatry.

[CR52] Zimmerman M, Galione JN, Chelminski I, Young D, Dalrymple K, Ruggero CJ (2010). Sustained unemployment in psychiatric outpatients with bipolar disorder: frequency and association with demographic variables and comorbid disorders. Bipolar Disord.

